# Prediction-Based Family Selection in Early Stage Sugarcane Breeding: Comparing BLUP, BLUE, Phenotypic Indices, and Machine Learning

**DOI:** 10.3390/plants15131980

**Published:** 2026-06-26

**Authors:** Farrag F. B. Abu-Ellail, Liping Zhao, Siqi Tang, Jiayong Liu, Li Yao, Peifang Zhao, Fenggang Zan

**Affiliations:** 1 State Key Laboratory of Tropical Crop Breeding, Sugarcane Research Institute, Yunnan Academy of Agricultural Sciences, Yunnan Key Laboratory of Sugarcane Genetic Improvement, Kaiyuan 661699, China; 2 Breeding and Genetics Department, Sugar Crops Research Institute, Agriculture Research Center, Giza 12619, Egypt

**Keywords:** sugarcane breeding, seedling stage, BLUP, BLUE, family selection, machine learning, LASSO, MFIDI, agreement indices, method comparison

## Abstract

Selecting superior families at the seedling stage is crucial for accelerating genetic gain in sugarcane, yet systematic comparisons of selection methods remain limited. This study evaluated seven selection strategies: phenotypic check-based selection (Pheno), a three-trait combined index (CI3), Best Linear Unbiased Prediction (BLUP), Best Linear Unbiased Estimation (BLUE), tiered family selection (Tiered), logistic regression (LASSO), and the Multi-Trait Family Ideotype Distance Index (MFIDI). The experiment followed an augmented block design with four blocks, two check varieties, and included 125 test families comprising 10,955 seedlings. Using a combined index of standardized cane and sugar yields, families were classified as elite (top 20%), moderate (60%), and weak (bottom 20%). BLUP and BLUE rankings were consistent (Spearman’s ρ > 0.95, TCI = 88%, Jaccard = 0.79). Elite families showed median index values of 0.90 (BLUP) and 0.88 (BLUE) with wide interquartile ranges, whereas weak families had medians of −0.70 with narrow ranges. LASSO achieved excellent predictive performance: AUC = 0.95, accuracy = 0.92, sensitivity = 0.90, specificity = 0.94, identifying cane yield, sugar yield, and millable cane as key drivers. Agreement for inferior families was lower across methods (BCI ≤ 68%). BLUP with a multi-trait index proved most effective for discriminating elite families. Families F31 and F71 consistently ranked top. Combining selection approaches with agreement indices improves early-stage decisions for family selection in sugarcane breeding.

## 1. Introduction

Accelerating genetic gain in the Yunnan sugarcane breeding program hinges on the early and accurate identification of superior sugarcane families (*Saccharum* spp.). However, the seedling stage presents significant challenges: large populations are evaluated in non-replicated rows, where environmental heterogeneity often masks true genetic potential, making selection inefficient, especially for low-heritability traits such as cane and sugar yield [1,2]. The urgency of this challenge is underscored by the fact that sugarcane supplies 85–90% of China’s domestic sugar, with Yunnan ranking as the second largest producer [1]. Breeding programs face persistent challenges, including insufficient planting material and lack of replication, all of which undermine selection accuracy [3,4]. Moreover, the number of seedlings per family often varies considerably due to differences in seed germination and establishment. Such unbalanced family sizes can bias selection decisions if not properly handled. Discarding families with few seedlings would sacrifice valuable genetic diversity, as these small families may carry superior alleles. Therefore, appropriate statistical methods are needed to handle unbalanced data while retaining all families [5,6,7]. For decades, mass (individual) selection has been the conventional approach, relying on visual scoring. However, it is inefficient for low heritability traits because environmental effects largely outweigh genetic variation [8,9]. This limitation has prompted a shift toward family selection, which uses replicated plots to improve precision and increase the probability of identifying superior clones while optimizing resources [2,5].

Family selection produces larger genetic gains than individual selection, and a two-stage approach (family followed by individual) is superior [5]. The effectiveness of family selection depends critically on the statistical method used to rank families. Traditional approaches include Best Linear Unbiased Prediction (BLUP) and Estimation (BLUE) [10]. Phenotypic indices and the Multi-trait Family Ideotype Distance Index (MFIDI) offer multi-trait frameworks [11,12]. Tiered selection effectively culls poor families [9,13], while simple combined indices (CI3) provide a computationally accessible alternative. More recently, machine learning methods like LASSO logistic regression have shown superior predictive accuracy, particularly for low heritability traits [14,15]. In plant breeding, LASSO has been successfully applied for genetic prediction and trait selection [2], while ideotype-based indices such as MFIDI have proven effective for multi-trait selection [12]. Despite the availability of these diverse tools, systematic comparisons at the seedling stage remain limited, especially in Chinese breeding programs. Moreover, rank consistency across methods can be quantified using agreement indices such as the Top Coincidence Index (TCI), Jaccard coefficient, and Bottom Coincidence Index (BCI) [7], yet such evaluations are scarce. High agreement indicates robustness in family rankings, whereas low agreement may indicate that methods capture different aspects of genetic merit or respond differently to data structure.

Most previous studies compared only two or three methods (e.g., BLUP vs. BLUE or mass selection vs. family selection) [9,10,13]. Consequently, there is a knowledge gap regarding how a wider range of approaches, from simple phenotypic indices to machine learning, perform under the same conditions, particularly when family sizes are highly variable and the design is unbalanced. To address this gap, the present study systematically compares seven family selection strategies—Pheno, CI3, BLUP, BLUE, Tiered, LASSO, and MFIDI—using seedling-stage data from 125 sugarcane families assessed for eight agronomic traits. These seven methods were selected to represent a spectrum from simple phenotypic indices (Pheno, CI3) through mixed-model approaches (BLUP, BLUE) and practical breeding pipelines (Tiered) to advanced machine learning (LASSO) and ideotype-based (MFIDI) selection.

The specific objectives are: (i) to compare the efficiency of these seven methods during the seedling stage; (ii) to quantify consistency among the seven methods using three agreement indices—the Top Coincidence Index (TCI) and Bottom Coincidence Index (BCI) for measuring overlap in the selected (top 20%) and rejected (bottom 20%) tails, together with the Jaccard coefficient for size-standardized global similarity; and (iii) to identify the approach(es) that provide the highest agreement and predicted genetic gain. By leveraging classical parametric models, tiered selection, machine learning, and ideotype-based indices, this study aims to enhance breeding efficiency, shorten the selection cycle, and increase the annual rate of genetic gain through the optimal use of phenotypic, estimated, and predicted information at the earliest stages.

## 2. Materials and Methods

### 2.1. Plant Material and Experimental Design

The study was conducted at the Yunnan Sugarcane Research Institute (YSRI), Yunnan Academy of Agricultural Sciences (YAAS), in Kaiyuan, Yunnan, China (approx. 23.72° N, 103.27° E; elevation ~1259 m a.s.l.), using 125 sugarcane families derived from elite germplasm produced from true seed (seedling propagation). The experiment was laid out in an augmented block design II [16], with four blocks. Two check varieties (Yun 0551 and ROC22) were replicated in every block. The 125 test families were assigned to only one block each and distributed across the four blocks according to the available number of seedlings per family, ensuring a workable plot size within each block while maintaining the augmented design structure. Within each block, families were planted in one or more rows of 8 m length with standard spacing (0.50 m between seedlings within a row and 1.20 m between rows). The actual number of seedlings per family varied according to seed germination and seedling establishment, ranging from 12 to approximately 200 seedlings per family across the four blocks. A total of 125 test families (comprising 10,955 seedlings) were evaluated across the four blocks. All agronomic practices followed standard recommendations for the region. A complete list of the 125 families, their codes (F1–F125), pedigree names, and the number of test seedlings per family is provided in Table 1. Throughout this manuscript, families are referred to by their codes.

### 2.2. Phenotypic Data Collection

Most data were recorded at maturity (12 months after transplanting). The following traits were measured or calculated:

- Surviving clumps: counted manually per row at 3 months after transplanting (before canopy closure), when clumps were easily distinguishable. Any clump that remained alive after transplanting from the nursery to the field was recorded as surviving. For families with multiple rows, each row contributed an independent observation; thus, all summary statistics refer to row-level averages, not family totals.
- Total plants per row were calculated as surviving clumps in that row × average number of stalks per clump, based on a random sample of at least 10 clumps from the same row (or all clumps if fewer than 10) [6].
- Stalk height (cm): measured from ground level to the top visible dewlap using a measuring tape on one representative stalk per sampled clump.
- Stalk diameter (cm): measured at the middle internode of the same stalk using a digital caliper.
- Millable stalks per hectare (ha^−1^): calculated as (millable stalks in sample plot/sample plot area in m^2^) × 10,000.
- Cane yield (t ha^−1^): calculated as follows: (total stalk weight in sample plot (kg)/number of millable stalks in sample plot) × (millable stalks ha^−1^/1000).
- Brix (%): was measured using a handheld ATAGO MASTER-T analog refractometer (ATAGO Co., Ltd., Tokyo, Japan) with a range 0.0–33.0% Brix, resolution 0.2%, accuracy ±0.2%, with automatic temperature compensation.
- Sucrose (%): estimated as: Brix (%) × 1.0825 − 7.703 [17].
- CCS (%): calculated using the formula [18] CCS (%) = {Sucrose (%) − [Brix (%) − Sucrose (%)] × 0.4} × 0.74.
- Sugar yield (t ha^−1^): calculated as (cane yield × CCS %)/100.

### 2.3. Statistical Analysis

#### 2.3.1. Augmented Block Design ANOVA

Analysis of variance (ANOVA) was conducted following the augmented block design with two checks replicated in each of four blocks, while the 125 test families were assigned to a single block each [16]. Total variation was partitioned into blocks, entries, and error. The entry sum of squares was subdivided into three orthogonal contrasts: (i) Checks (variation between the two checks), (ii) Test families (variation among test families), and (iii) Checks vs. Test (mean of checks vs. mean of test families). The ANOVA structure and expected mean squares are presented in Table 2. All calculations were performed using R Software, Core Team [19].

#### 2.3.2. Mixed Model Analysis (REML/BLUP)

The augmented design is inherently unbalanced due to unreplicated test families and variable plot sizes. Accordingly, a linear mixed model was fitted using Restricted Maximum Likelihood (REML). The model was

y_ij_ = μ + B_i_ + F_j_ + ε_i__j_

where y_i__j_ is the phenotypic observation of family j in block i, μ is the overall mean, B_i_ is the fixed effect of block i (i = 1… 4) F_j_ is the random effect of family j (j = 1… 125), and ε_i__j_ is the residual error. Variance components were estimated using REML, and Best Linear Unbiased Predictions (BLUPs) for family effects were obtained from the mixed model equations. Broad-sense heritability on a family-mean basis was estimated directly from the variance components as

h^2^ = σ^2^g/(σ^2^g + σ^2^e).

where σ^2^g is the genotypic variance and σ^2^e is the residual variance. Genetic advance as a percentage of the mean (GA%) was computed as

$$GA\%=(i\times \sigma p\times h^{2})/\bar{X}\times 100.$$

where i = 1.4 (selection intensity of 20%), σp is the phenotypic standard deviation, and $\bar{X}$ is the grand mean of each trait. This unified approach accounts for the single replication of test families, variable seedling numbers, and block effects [6,7]. The REML/BLUP analysis served as the primary framework for estimating variance components, heritability, and family-wise BLUPs, upon which all subsequent results (family ranking, selection gains, and agreement indices) are based. The genotypic coefficient of variation (CVg) and the environmental coefficient of variation (CVe) were calculated for each trait using the following formulas:

CVg (%) = (√σ^2^g/μ) × 100

CVe (%) = (√σ^2^e/μ) × 100

where σ^2^g is the genotypic variance, σ^2^e is the residual variance, and μ is the overall mean of the trait (calculated from the raw data of test families). These coefficients provide standardized measures of the relative magnitude of genetic and environmental variation, allowing comparison across traits measured on different scales.

#### 2.3.3. Validation of Ranking Stability

Two validation analyses were performed using R software (version 4.6.0; R Core Team, 2026). (i) A sensitivity analysis removed all families with fewer than 40 seedlings (42 families), and the multi-trait index (MTI) was recalculated for the remaining 83 families.

(ii) Bayesian linear mixed models were fitted using the “blme’ package (default Wishart prior) and compared with standard REML models (‘lme4’ package). Spearman’s rank correlations were calculated using base R.

### 2.4. Selection Methods

Seven selection methods were compared:

#### 2.4.1. Phenotypic Means Check-Based Selection (Pheno)

Rationale: Provides a simple, check-based benchmark that reflects current breeding practice; families are compared directly to the best-performing check variety across all traits. For each of the 125 test families and for each of the eight agronomic traits, the family mean was compared to the best check value (the maximum of ROC22 and Yun 0551). Three categorical outcomes were recorded for each trait: “greater than”, “equal to”, or “less than” the best check. A categorical comparison matrix (rows = families, columns = traits) was then constructed for the top-ranking families (based on an initial multi-trait index for sorting only) and visualized as a heatmap. The heatmap uses a color scale (green = greater than, yellow = equal to, red = less than) to display the performance of each family relative to the best check. This approach allows rapid identification of families that outperform the checks in specific traits without discarding any family based on a single count.

#### 2.4.2. Combined Index-Based Three-Trait Selection (CI3)

Rationale: Offers a simple multi-trait score without complex variance modeling, balancing cane yield, sugar yield, and millable cane. To integrate three key yield components (cane yield, sugar yield and millable cane) into a single selection criterion, a combined index was computed for each family. For any given method of obtaining family-level estimates (e.g., raw phenotypic means, BLUP or BLUE), let *CYi*, *SYi* and *MCi* denote the values for cane yield, sugar yield, and millable cane for the *i*-th family. The combined index *i* was calculated as the average of the standardized values

$$Ii=\frac{1}{3}\left(\frac{CYi-\bar{CY}\;}{{SD}_{CY}}\right)+\left(\frac{\left(SYi-\bar{SY}\right)}{{SD}_{SY}}\right)+\left(\frac{MCi-\bar{MC}}{{SD}_{MC}}\right)\;$$

where $\bar{CY}$, $\bar{SY}$, $\bar{MC}$, *SD_CY_*, and *SD_MC_* are the overall means and standard deviations of the respective traits across all families. Families were then ranked in descending order of *i*. Based on this ranking, the top 20% of families were classified as “Good,” the bottom 20% as “Poor,” and the remaining 60% as “Intermediate”.

#### 2.4.3. Selection Based on a BLUP and BLUE Combined Index

Rationale: Compares the two standard mixed-model estimators (BLUP = random effects, BLUE = fixed effects) to assess their agreement and to justify using BLUP as the primary method for unbalanced data. Family level BLUPs and BLUEs for sugar yield and cane yield were obtained from the mixed model described in Section 2.3.2. A simplified combined index was constructed as the average of the standardized values of these two traits. Standardization was performed by subtracting the overall mean and dividing by the overall standard deviation. Families were ranked in descending order of their index values. Based on the BLUP index distribution, families were classified into three categories: elite, moderate, and weak. The top 10 families were retained for detailed comparison between BLUP and BLUE, while scatter plots assessed agreement across all families.

#### 2.4.4. Tiered Family Selection (Tiered)

Rationale: Mimics a practical, step-down breeding pipeline where selection intensity increases with family merit, saving resources by discarding inferior families early. Genotypic values for cane yield (t ha^−1^) were obtained from mixed model analysis. Families were ranked and grouped into percentiles. Differential within-family selection intensities were applied following a tiered (step-down) strategy: from the 10 families above the 90th percentile, 40% were retained; from the 10 families between P80–P90, 30%; from the 10 families between P70–P80, 20%; from the 10 families between P60–P70, 10%; while the 85 families below P60 were discarded (0%). This approach follows [9,13]. The cumulative effect was visualized using a step-down selection curve.

#### 2.4.5. Machine Learning Method: LASSO Logistic Regression

Rationale: Identifies the most informative traits for distinguishing elite families using built-in regularization, reducing overfitting and improving reproducibility. A logistic regression with L1 regularization (LASSO) was used to classify families as “Good” (top 20% based on MTI) vs. “Other” (remaining 80%). All eight agronomic traits were entered as predictors. The dataset was randomly split into training (70%) and testing (30%) sets. The optimal regularization parameter λ was selected via 10-fold cross-validation on the training set using the cv.glmnet function [15]. The final model was evaluated on the held-out test set using area under the curve (AUC), accuracy, sensitivity, and specificity. Families were ranked by their predicted probability of being “Good”, and the top 20% were considered selected by this method. Overfitting is mitigated by the L1 penalty (which performs automatic feature selection) and by the cross-validated choice of λ.

#### 2.4.6. Multi-Trait Family Ideotype Distance Index (MFIDI)

Rationale: Ranks families by their proximity to an ideal genotype (ideotype) in a reduced multi-trait space, enabling simultaneous improvement of several traits without arbitrary weighting. To combine six important yield-related traits (stalk height, stalk diameter, Brix, millable cane per hectare, cane yield, and sugar yield) into a single selection criterion, the MFIDI was computed using the metan package in R Software Core Team [19]. The ideotype was defined as 105% of the maximum observed value for each trait (all traits were desirable at higher values). The MFIDI was calculated as the Euclidean distance between each family and the ideotype in a reduced factor space derived from factor analysis with varimax rotation. Families were ranked by MFIDI in ascending order (lower distance = better), and the top 20% (i.e., families with the smallest distances) were designated as selected. A factor analysis biplot was generated to visualize trait contributions and family distribution.

### 2.5. Agreement Indices Among Selection Methods

Rationale: TCI and BCI quantify the overlap in the extreme tails of the ranking (top 20% and bottom 20%), which are directly relevant for selection decisions. Jaccard provides a size-standardized measure of overall set similarity. Together, these three indices capture agreement from complementary perspectives. For each of the seven selection methods, the top 20% (25 best families) and bottom 20% (25 worst families) were identified. Let A_top and B_top denote the top 20% sets of two methods, A and B, and A_bottom and B_bottom their bottom 20% sets. Pairwise agreement was quantified using three indices

Top Coincidence Index (TCI) = (A_top ∩ B_top)/25 × 100;

Jaccard coefficient (top 20%) = (A_top ∩ B_top)/(A_top ∪ B_top) × 100;

Bottom Coincidence Index (BCI) = (A_bottom ∩ B_bottom)/25 × 100.

These indices were calculated for all pairwise combinations of the seven methods (Pheno, CI3, BLUP, BLUE, Tiered, LASSO, and MFIDI). Results were visualized using heatmaps (for TCI, Jaccard, and BCI) and a faceted dot plot (for TCI) using R packages ggplot2, reshape2, dplyr, and tidyr by the R Software Core Team [19].

## 3. Results

### 3.1. Statistical Analysis

#### 3.1.1. Analysis of Variance (Augmented Block Design)

Analysis of variance based on the augmented block design (Table 3) revealed highly significant block effects for all traits (*p* < 0.01), confirming that the four blocks effectively accounted for spatial heterogeneity. The overall entry (families) effect was significant for total plants, stalk height, stalk diameter, millable cane, cane yield, and sugar yield (*p* < 0.05 or *p* < 0.01); however, it was not significant for surviving clumps (*p* = 0.073) or Brix (*p* = 0.051). Furthermore, partitioning the entry sum of squares showed that the subset of test families exhibited significant variation for all traits (*p* < 0.05 or *p* < 0.01), indicating considerable genetic diversity among the 125 test families. The two check varieties did not differ significantly from each other for any trait (all *p* > 0.05), except for total plants, where the check difference was significant (*p* = 0.0032). The contrast between checks and test families was significant for all traits except total plants (*p* = 0.293), indicating that test families, on average, did not differ from the checks in plant population. Notably, for Brix, the contrast was significant (*p* = 0.012), suggesting genetic differences in sugar content between test families and checks. Augmented ANOVA confirms significant genetic variation, but due to an unbalanced design, REML/BLUP was used to obtain unbiased genetic parameters; these results form the basis of the interpretations.

#### 3.1.2. Mixed Model Analysis (REML/BLUP)

The augmented block design combined with REML/BLUP adjusts for the bias that would arise from unequal family sizes. Families with fewer seedlings have their estimates ‘shrunk’ toward the population mean, while families with more seedlings receive greater weight. This approach ensures that selection is based on genetic merit, not on family size. According to Table 4, genotypic variance (σ^2^g) was highest for millable cane (352.62), total plants per row (225.68), cane yield (266.82), and stalk height (177.05), indicating substantial genetic variation for yield-related traits. Conversely, low genotypic variances were observed for surviving clumps per row (2.69), stalk diameter (0.04), and Brix (0.82). Similarly, residual variances (σ^2^e) followed a comparable pattern, with the largest values recorded for total plants (194.52), cane yield (316.01), and stalk height (567.80), suggesting moderate-to-high environmental influence on these traits. Broad-sense heritability (h^2^%) on a family-mean basis ranged from 23.8% (stalk height) to 53.7% (total plants per row and millable cane). Intermediate estimates were obtained for surviving clumps (24.1%), Brix (37.4%), sugar yield (38.4%), stalk diameter (44.4%), and cane yield (45.8%). These values indicate moderate genetic control and a moderate response to selection. In addition, genetic advance as a percentage of the mean (GA%) followed similar trends: the highest expected gains were observed for total plants per row (34.9%), millable cane (34.9%), cane yield (30.5%), and sugar yield (26.3%), whereas stalk height (4.7%) and surviving clumps (12.8%) showed the lowest expected progress under selection. In summary, productivity-related traits (total plants, millable cane, cane yield, sugar yield) exhibited moderate heritability and moderate to high genetic advance, thereby supporting the feasibility of family-based selection in this population. The genotypic coefficient of variation (CVg) ranged from 4.56% (Brix) to 26.63% (millable cane), while the environmental coefficient of variation (CVe) ranged from 5.90% (Brix) to 31.59% (sugar yield). These values indicate moderate to high genetic variability for yield-related traits and confirm that the phenotypic variation is largely under genetic control.

### 3.2. Validation of Ranking Consistency

Two additional analyses were performed to assess the stability of the family rankings under alternative analytical choices.

#### 3.2.1. Effect of Removing Families with Fewer than 40 Seedlings

**Figure 1:**
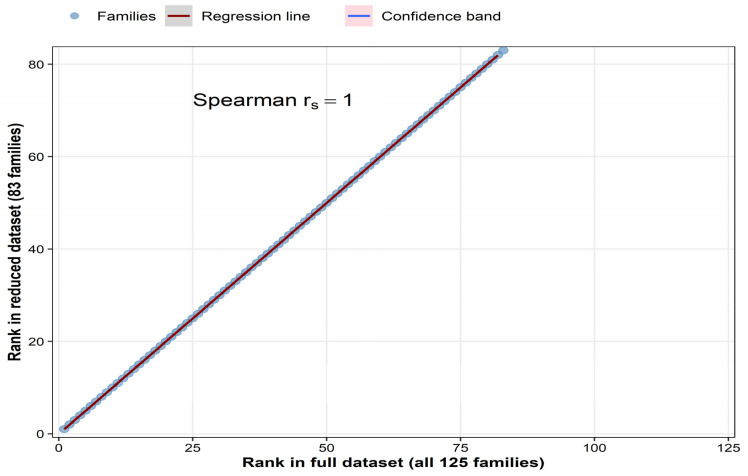
Sensitivity analysis: Scatter plot of MTI-based rankings for all 125 families (x-axis) versus the reduced set of 83 families (y-axis) after removing families with fewer than 40 seedlings. Each point represents a family.

A sensitivity analysis was conducted by removing all families with fewer than 40 seedlings (42 families, 33.6% of the total) and recalculating the multi-trait index (MTI) for the remaining 83 families. The Spearman rank correlation between the original ranking (125 families) and the reduced ranking was perfect (r_s_ = 1.00; Figure 1). This demonstrates that the presence of small families does not bias the relative order. Therefore, their removal is statistically unjustified and would not improve selection accuracy; instead, it would only discard valuable genetic diversity. Retaining all families is therefore both scientifically justified and essential for capturing the full genetic potential of the breeding population.

#### 3.2.2. Comparison of REML and Bayesian BLUP

**Figure 2:**
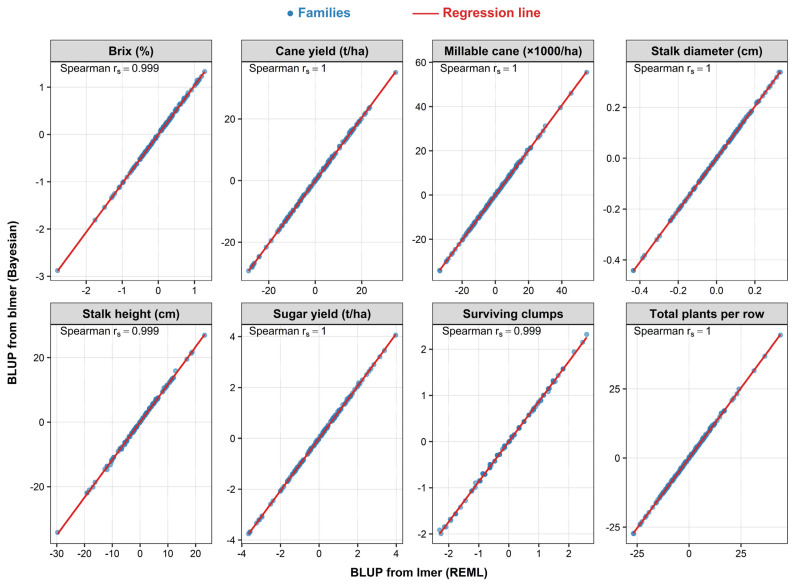
Scatter plots of family BLUPs from the REML (lmer) and Bayesian (blme) models. Blue points: families; red line: linear regression fit. Axes titles indicate the estimation method. Spearman rank correlation coefficients (r_s_) are displayed inside each facet (values ≥ 0.999 for all eight traits).

Figure 2 presents the comparison between standard REML models (lmer) and Bayesian linear mixed models (fitted using the blme package in R with default Wishart prior), which was performed to evaluate whether potential over-shrinkage could affect the stability of family rankings. Family BLUPs were obtained from both methods for all eight traits: Surviving clumps, total plants per row, stalk height, stalk diameter, Brix, millable cane, cane yield, and sugar yield. The Spearman rank correlations between the two sets of BLUPs were ≥0.999 for every trait (0.999 for surviving clumps, total plants, stalk height and Brix; 1.000 for stalk diameter, millable cane, cane yield and sugar yield; Figure 2). This near-perfect agreement demonstrates that the family rankings are highly stable and not sensitive to the choice of estimation method. Consequently, the additional complexity of a full Bayesian analysis is not required to support our conclusions.

### 3.3. Selection Methods

Seven selection methods were compared.

#### 3.3.1. Phenotypic Check-Based Selection (Pheno)

##### Distribution of Test Full Families

**Figure 3:**
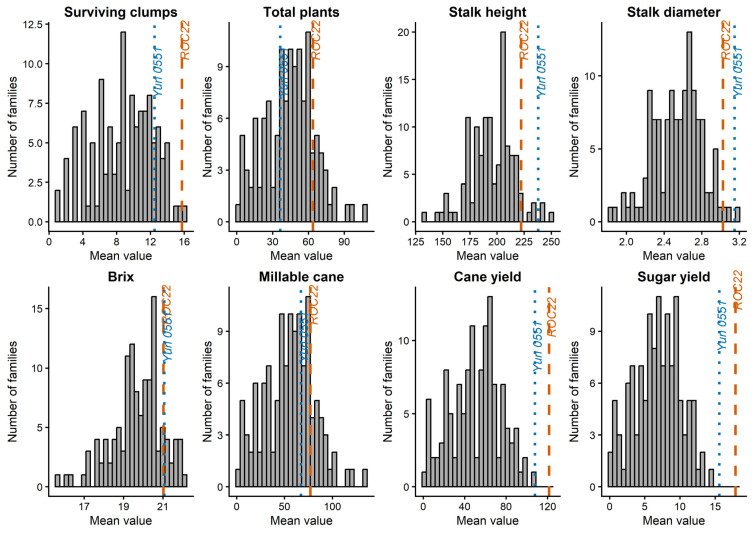
Distribution of eight agronomic and quality traits among 125 sugarcane families (*n* = 125) compared with the best check values. Histograms show family means; vertical lines indicate the best values of the two checks (orange dashed: ROC22; blue dotted: Yun 0551).

The distribution of the 125 test families for each trait, together with the best check values as reference lines, is visualized in Figure 3. Specifically, for surviving clumps and total plants, the distributions were strongly left-skewed: most families fell below both check varieties, and only a few approached or exceeded the best check. A similar pattern was observed for stalk height and stalk diameter, although these traits exhibited wider variation, with a small subset of families surpassing the best check values. By contrast, Brix showed a distribution centered near the check values; several families reached or slightly exceeded the best check, indicating potential for quality improvement. However, cane yield and sugar yield displayed highly skewed distributions, with the vast majority of families performing substantially below the best check. Notably, for these yield-related traits, virtually all test families fell to the left of both check lines. Finally, the categorical comparison against the best check revealed that 26 test families (20.8%) outperformed the best check in at least one trait, while only one family (F85) exceeded the best check in three traits (total plants, stalk height, and millable cane). Consequently, for the remaining 99 test families (79.2%), all eight traits were inferior to the best check.

##### Comparison Matrix for Top Families

**Figure 4:**
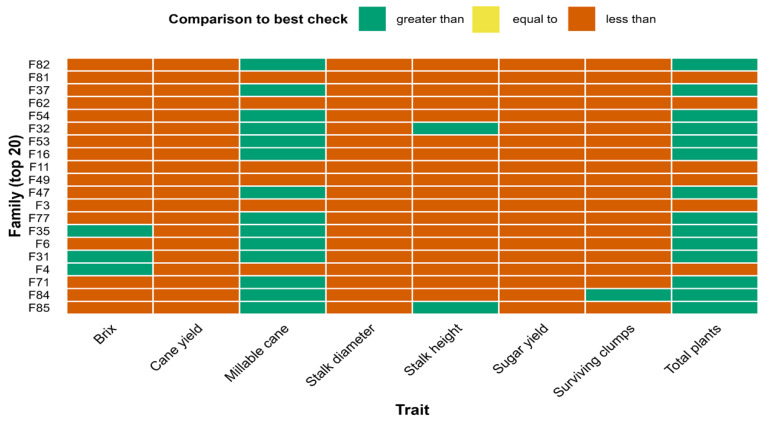
Heatmap of the categorical comparison matrix for the top 20 test families (ranks 3–22) and the two check varieties. Families (rows) are ordered by their multi-trait index rank (highest to lowest). Check varieties are indicated with “(Check)” and separated from test families by a horizontal facet. Colors: green = greater than best check, yellow = equal to, red = less than.

Figure 4 presents the comparison matrix for two checks (ROC22 and Yun 0551) and the top 20 test families (ranks 3–22), ordered by descending combined index. The two checks occupy the top two rows with mostly “equal to” and “less than” entries. Among the test families, “greater than” cells are few and concentrated in total plants, stalk height, and millable cane. Specifically, family F85 leads with three “greater than” entries (total plants, stalk height, millable cane), and families F84, F31, F35, and F32 also show three each. In addition, a larger group (F71, F6, F77, F47, F16, F53, F54, F37, and F82) has two “greater than” entries each, while family F4 has only one. By contrast, families F3, F49, F11, F62, and F81 have none. Thus, the heatmap effectively discriminates top families, supporting the combined index for early-stage sugarcane breeding.

#### 3.3.2. CI3-Combined Index Selection

##### Frequency Distribution of CI3 Values

**Figure 5:**
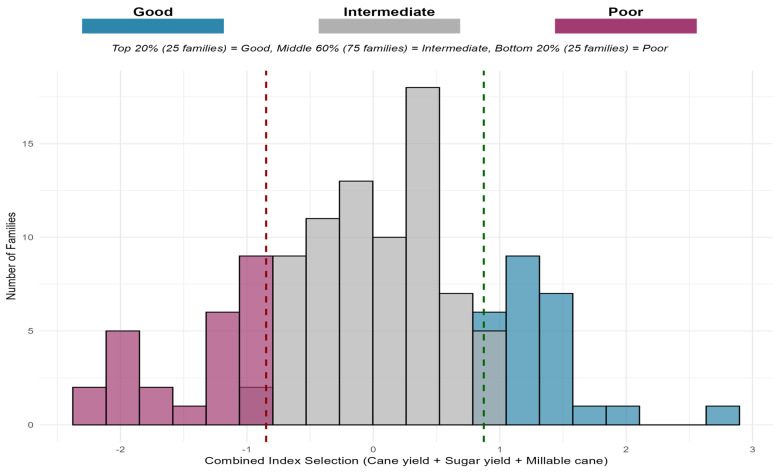
Frequency distribution of the CI3 combined index for 125 sugarcane families (F1–F125). The index was calculated as the average of standardized cane yield, sugar yield, and millable cane. Histogram bins are 0.25 wide. Families are color-coded according to their performance class: Good (dark sky blue, I ≥ 0.87, top 20%, *n* = 25); Intermediate (gray, 0.87 > I > −0.85, middle 60%, *n* = 75), and Poor (light purple, I ≤ −0.85, bottom 20%, *n* = 25). Vertical dashed lines mark the classification thresholds.

The CI3 (Combined Index of three traits) was computed for each of the 125 sugarcane families as the average of the standardized values of cane yield, sugar yield, and millable cane, following the procedure described in Section 2.4.2. Based on the ranking, families were classified into three groups: Good (top 20%, I ≥ 0.87), Intermediate (middle 60%, 0.87 > I > −0.85), and Poor (bottom 20%, I ≤ −0.85). Figure 5 shows the frequency distribution of the CI3 index, which ranges from −2.25 (F59) to 2.76 (F71). Notably, the histogram is approximately bell-shaped with a slight negative skew, indicating that most families clustered near the mean, while a smaller number showed extreme performance. Vertical dashed lines mark the classification thresholds (Good: *n* = 25, Intermediate: *n* = 75, Poor: *n* = 25). Consequently, this distribution confirms that CI3 successfully differentiates superior from inferior families.

##### Top Ten and Bottom Ten Families Based on CI3

Table 5 presents the ten highest-ranking (Good) and ten lowest-ranking (Poor) families in a single table for direct comparison. The top performer, F71, attained an index of 2.76, driven by exceptionally high Z_Millable (3.54) and Z_Cane (2.70). The second-ranked family, F31, achieved an index of 1.93, with the highest Z_Sugar (2.59) among all entries. Notably, all top ten families exhibited consistently positive Z-scores across the three traits, indicating either balanced or outstanding performance. In contrast, the ten lowest-ranking families all had negative index values and negative Z-scores for all three traits. The poorest family, F59, had an index of −2.25 and the lowest Z_Cane (−2.23), Z_Sugar (−2.35), and Z_Millable (−2.16).

#### 3.3.3. BLUP and BLUE Combined Index

BLUP and BLUE estimates were computed for the eight traits across all 125 families. Notably, Spearman’s rank correlations exceeded 0.95 for every trait, indicating strong agreement between the two estimators. To obtain a single selection criterion, a simplified combined index was calculated as the average of standardized sugar yield and cane yield values. Based on the BLUP index distribution, families were then classified into three performance groups: elite (top 20%), moderate (60%), and weak (bottom 20%).

##### BLUP and BLUE-Based Family Classification

**Figure 6:**
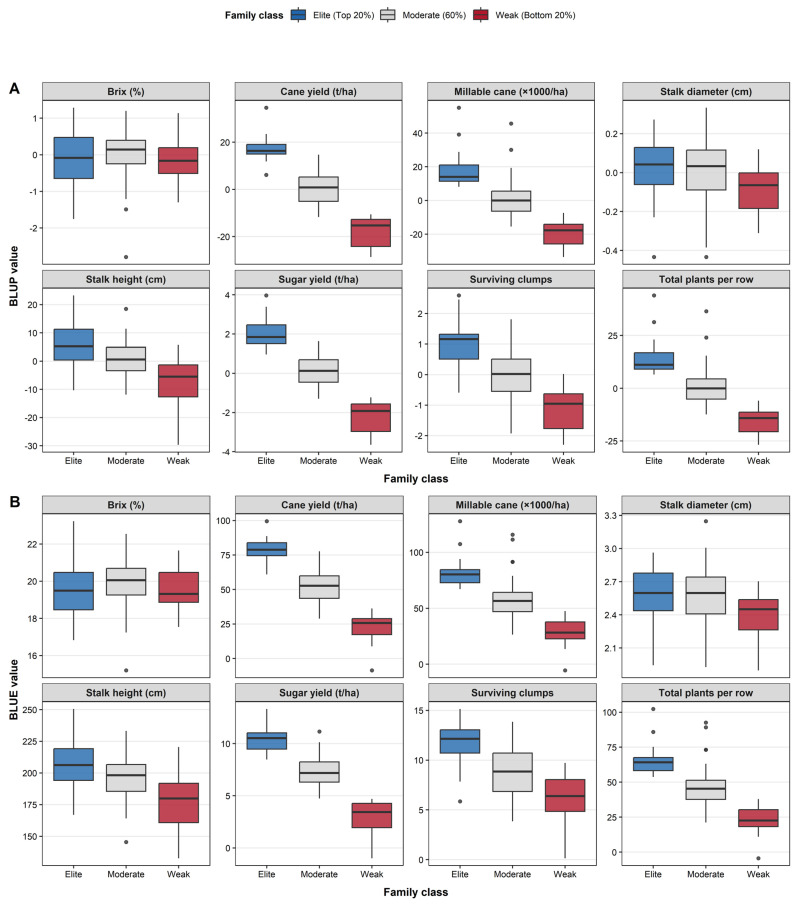
Boxplots of BLUP (**A**) and BLUE (**B**) values for eight agronomic traits in 125 sugarcane families. Families are classified into elite (dark blue), moderate (gray), and weak (brick red) based on a combined index of cane and sugar yields (top 20%, 60%, and bottom 20%, respectively). Boxes show the interquartile range (IQR), horizontal lines the median, and whiskers extend to 1.5 × IQR. The x-axis shows the three classes (1 = elite, 2 = moderate, 3 = weak) without labels to avoid clutter; the y-axis presents BLUP/BLUE values on a trait-specific scale.

Looking first at the BLUP results (Figure 6A), the largest differences between elite and weak families were observed for total plants per row (median 21.58 vs. −5.87), millable cane (26.98 vs. −7.33), and cane yield (21.28 vs. −7.87). In contrast, stalk diameter (0.18 vs. −0.09) and Brix (0.82 vs. −0.47) showed much narrower separation, suggesting less pronounced genetic contrast for these two traits. Regarding the top-performing families, the ten highest-ranking families (by BLUP index) were F31 (index 1.85), followed by F71, F4, F16, F35, F3, F103, F77, F6, and F63. Among these, F71 achieved the highest cane yield (34.70 t ha^−1^) and millable cane (55.13 × 1000 ha^−1^), whereas F31 recorded the highest sugar yield (3.98 t ha^−1^), and F63 exhibited the highest Brix (1.29%). Furthermore, across all 125 families, BLUP values ranged widely: from −28.71 to 34.70 for cane yield, from −33.55 to 55.13 for millable cane, and from −3.66 to 3.98 for sugar yield. Taken together, these results confirm broad genetic diversity for family-based selection.

Turning to the BLUE results (Figure 6B), elite families consistently outperformed moderate and weak families across all traits, with the most pronounced differences observed for yield-related variables. Specifically, the median BLUE values for elite, moderate, and weak families were Brix 21.1%, 20.3%, and 18.9%; cane yield 85.0, 65.0, and 45.0 t ha^−1^; millable cane 95.0, 75.0, and 55.0 (×1000 ha^−1^); and sugar yield 10.5, 8.0, and 6.0 t ha^−1^, respectively. Among the top ten families based on field measurements, F31 recorded the highest sugar yield (14.14 t ha^−1^) and Brix (21.68%); F71 achieved the highest millable cane (134.17) and cane yield (106.80 t ha^−1^); F4 followed with a sugar yield of 13.25; F16 produced 113.75 millable cane; F35 gave a 12.17 sugar yield and 21.31% Brix; and F63 exhibited the highest Brix (22.1%). Across all 125 families, the BLUE ranges were 18.35–106.80 t ha^−1^ for cane yield, 1.25–134.17 for millable cane, and 0.12–14.14 t ha^−1^ for sugar yield. Thus, the BLUE boxplots confirm that the combined index effectively discriminates superior families.

##### BLUP vs. BLUE Index Comparison

**Figure 7:**
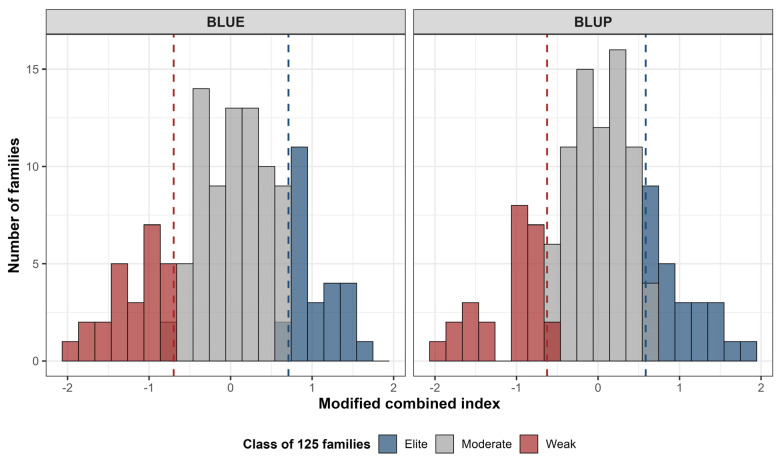
Distribution of the combined index for 125 sugarcane families estimated by BLUP (**left**) and BLUE (**right**). Families are color-coded based on BLUP rankings: elite (top 20%) in blue, moderate (60%) in gray, and weak (bottom 20%) in red.

Figure 7 compares the distribution of the combined index for BLUP and BLUE. Among the 25 elite families identified by BLUP, 15 had index values above 1.0 (with one reaching 1.6–1.8). In contrast, BLUE placed only 14 above 1.0 (none >1.6). This difference reflects BLUP’s shrinkage property: it is conservative for less consistent families while slightly amplifying the merit of top-ranking ones. For index values below zero, both methods gave nearly identical distributions. The moderate class (−0.6 to 0.6) peaked at 0.0–0.2 with 18 families in each method. Similarly, the weak class (<−0.6) had eight families in the −1.0 to −0.8 bin for both. Therefore, BLUP and BLUE agree on the central and lower parts of the distribution. However, BLUP better discriminates elite families, showing a more right-skewed distribution with higher elite values, making it more suitable for early-stage selection. Nevertheless, BLUE remains a valid alternative when computational resources are limited.

#### 3.3.4. Tiered Family Selection

**Figure 8:**
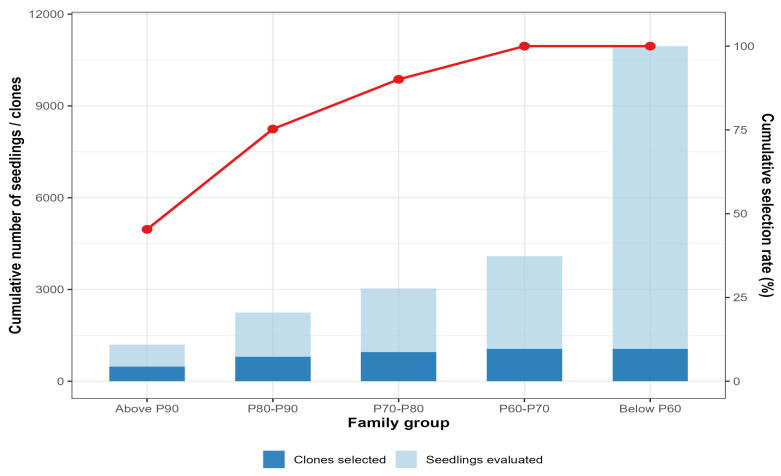
Step-down selection curve showing cumulative number of seedlings evaluated (light blue bars) and clones selected (dark blue bars) across five family performance groups. The red line and points represent the cumulative selection rate (%), shown on the secondary y-axis.

As illustrated in Figure 8, our evaluation of 125 families (comprising 10,955 seedlings) under tiered selection yielded the following results. After applying differential within-family selection intensities, we selected 1059 clones overall, corresponding to 9.67% of the total seedlings. Specifically, our results show that the 20 families above the 80th percentile contributed 797 selected clones, which accounted for 75.3% of all selected clones. In contrast, the 85 families below the 60th percentile (representing 68% of all families) were completely discarded; these families comprised 6869 seedlings (62.7% of the total) and yielded no selected clones.

The data further indicate that the cumulative number of evaluated seedlings increased steadily across groups, whereas the number of selected clones plateaued after the P60–P70 group because no further selections were made from lower-performing families. Consequently, the cumulative selection rate rose rapidly in the top groups, reaching a final value of 9.67%. These findings clearly demonstrate the efficiency of the strategy: most selected clones originated from the top 20% of families (those above P90 and P80–P90). Overall, directing selection intensity toward the top 20% of families captured more than three-quarters of all selected clones, while eliminating the bottom 68% of families substantially reduced operational costs without sacrificing genetic gain.

#### 3.3.5. LASSO Machine Learning Predictive Performance

##### Cumulative Logistic Regression Patterns

**Figure 9:**
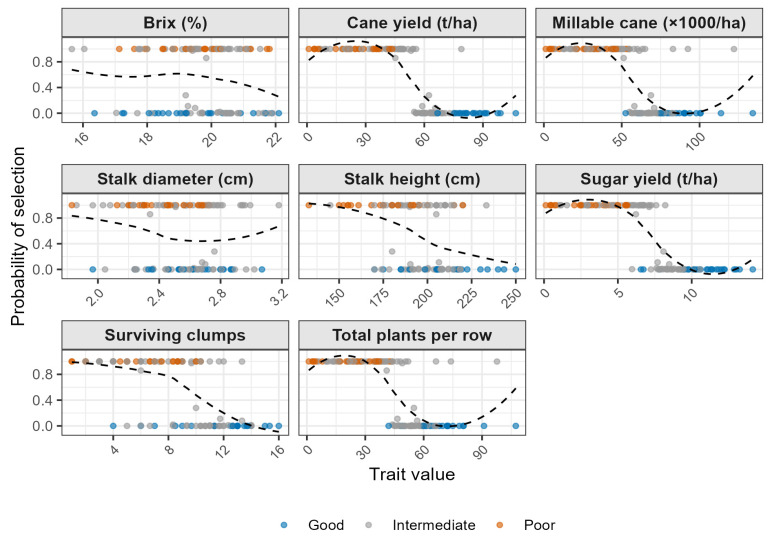
LASSO machine learning-based cumulative logistic regression curves for eight agronomic traits across 125 sugarcane families. Color coding reflects family classification: blue (Good, top 20%), gray (Intermediate, middle 60%), and orange (Weak, bottom 20%).

Figure 9 presents the cumulative logistic regression curves for eight traits across 125 families (10,955 seedlings), revealing distinct classification patterns. The curves plot the predicted probability of a family being “Good” (top 20% genotypic) against standardized trait values, grouping traits into three categories. Specifically, cane yield, sugar yield, and millable cane exhibited steep sigmoidal patterns, rising sharply from low to high probability over a narrow standardized range, thereby confirming them as key drivers of family classification. For instance, when standardized cane yield exceeded 0.6, the predicted probability of being “Good” was above 80%. In contrast, Brix and stalk diameter produced flat curves, with probabilities remaining near 0.5 across most values, indicating limited utility for classification. Conversely, stalk height showed a slight downward trend, consistent with its negative LASSO coefficient. Meanwhile, surviving clumps and total plants per row displayed intermediate, noisier patterns, reflecting moderate discriminative ability. Thus, yield-related traits (cane yield, sugar yield, and millable cane) are the primary drivers of successful classification, whereas quality-related traits (Brix, stalk diameter) contributed little at this selection stage.

##### Model Performance on the Test Set

The LASSO model was evaluated on a hold-out test set of 25 families (20% of 125). As reported in Table 6, it achieved an AUC of 0.95, a classification accuracy of 0.92, a sensitivity of 0.90, and a specificity of 0.94. To begin with, AUC values between 0.91 and 1.00 are regarded as excellent, confirming reliable discrimination between “Good” families and the rest. In detail, the high specificity (0.94) indicates that 94% of poor families were correctly classified as “Remaining”, thereby minimizing the risk of advancing inferior material. On the other hand, the sensitivity (0.90) shows that 90% of true “Good” families were correctly identified, missing only 10% of superior families. As a result, these balanced metrics reflect an optimal trade-off across classification thresholds. In conclusion, the LASSO model proves reliable for early-generation family classification in sugarcane breeding.

#### 3.3.6. Multi-Trait Family Ideotype Distance Index (MFIDI)

**Figure 10:**
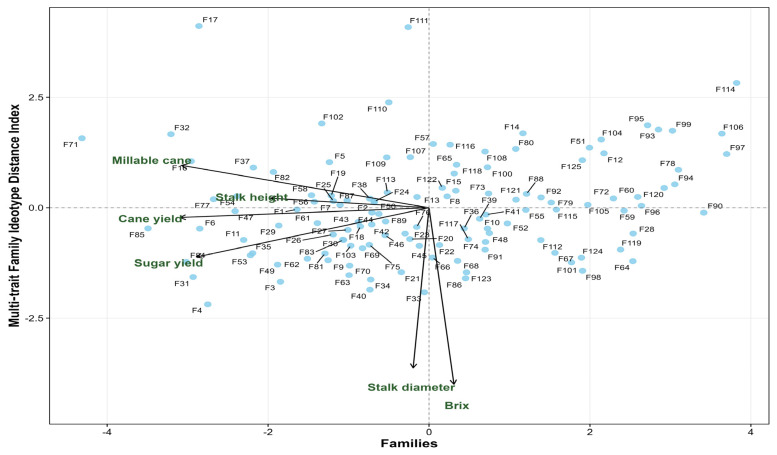
Factor analysis biplot of 125 sugarcane families based on six agronomic traits. Points (sky blue) represent families labeled with codes (F1–F125). Black arrows indicate trait contributions; trait labels are in dark green. The top five MFIDI families (F4, F31, F84, F3, and F53) appear in the positive quadrants. The first two factors explain 73.9% of the total variance.

MFIDI was computed for 125 families based on six traits: stalk height, stalk diameter, Brix, millable cane, cane yield, and sugar yield (Figure 10). Specifically, the ideotype was defined as 105% of the maximum observed value for each trait, and MFIDI was calculated as the Euclidean distance between each family and the ideotype in a reduced factor space derived from factor analysis with varimax rotation. Thus, lower MFIDI values indicate closer proximity to the ideotype and, consequently, superior performance. In this study, MFIDI values ranged from 1.33 to 6.46 (mean = 3.60, SD = 1.03). Accordingly, the 25 families with the lowest MFIDI (top 20%) were selected as the most promising. Among them, the top five families were F4 (1.33), F31 (1.50), F84 (1.67), F3 (1.93), and F53 (2.03). Regarding the factor analysis, the varimax rotation generated a biplot (Figure 10), where the first two factors explained 73.9% of the total variance. Notably, Factor 1 (horizontal) was associated with cane yield, sugar yield, and millable cane, whereas Factor 2 (vertical) was associated with stalk diameter and Brix. As shown in the biplot, the top five families lie in the positive quadrants of both factors, thereby confirming their superiority across yield and quality-related traits. Moreover, families with lower MFIDI plot closer to the trait vectors, indicating balanced improvement. Finally, yield-related traits cluster together, while stalk diameter and Brix form a separate cluster, suggesting relative independence and thus enabling simultaneous selection for yield and sugar content. The overall ranking of all 125 families based on the synthesis of the seven selection methods is provided in Supplementary Table S1.

### 3.4. Agreement Among Selection Methods

**Figure 11:**
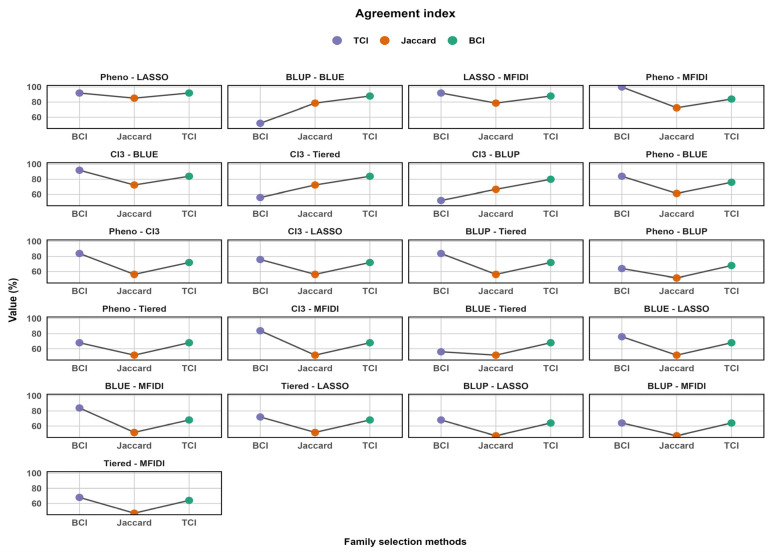
Pairwise agreement indices among seven sugarcane family selection methods (Pheno, CI3, BLUP, BLUE, Tiered, LASSO, MFIDI). Each panel represents a pair of methods and displays three indices: the Top Coincidence Index (TCI, green circles), the Jaccard index (orange circles), and the Bottom Coincidence Index (BCI, purple circles). TCI and Jaccard are based on the top 20% families; BCI is based on the bottom 20% families. Higher values indicate stronger agreement. The horizontal axis shows the three indices; the vertical axis gives the agreement value (%).

Pairwise agreement among the seven methods (Pheno, CI3, BLUP, BLUE, Tiered, LASSO, and MFIDI) was assessed using the Top Coincidence Index (TCI) and Jaccard coefficient for the top 20% families, as well as the Bottom Coincidence Index (BCI) for the bottom 20% (Figure 11). Regarding top-family agreement, the highest TCI values were observed between Pheno and LASSO (92.0%), followed by Pheno-MFIDI (84.0%) and BLUP-BLUE (88.0%). The Jaccard coefficients followed a similar pattern: Pheno-LASSO (85.2%), Pheno-MFIDI (72.4%), and BLUP-BLUE (78.6%). Thus, phenotypic checks strongly agree with machine learning approaches, while BLUP and BLUE rank top families almost identically. Using BLUP as a reference (widely adopted in breeding), BLUE exhibited the highest TCI (88.0%), followed by CI3 (80.0%) and Tiered (72.0%). In contrast, Pheno, LASSO, and MFIDI had lower TCI values (68.0%, 64.0%, and 64.0%, respectively). Similarly, the Jaccard coefficients relative to BLUP were BLUE (78.6%), CI3 (66.7%), Tiered (56.3%), Pheno (51.5%), LASSO (47.1%), and MFIDI (47.1%). Notably, all Jaccard values were lower than their corresponding TCI values, reflecting that disagreement leads to a union that exceeds the fixed top 20 set size (25 families).

Turning to the bottom 20% families (BCI), the highest agreement with BLUP was achieved by Tiered (84.0%), followed by LASSO (68.0%) and Pheno (64.0%). In contrast, BLUE and CI3 had BCI values of 52.0%, while MFIDI reached 64.0%. Consequently, Tiered’s high BCI confirms its effectiveness at identifying poor performers, consistent with discarding families below the 60th percentile. However, the low BCI of BLUE and CI3 suggests less stable rankings among the weakest families. Regarding the lowest TCI values, BLUP-LASSO (64.0%), BLUP-MFIDI (64.0%), and CI3-MFIDI (68.0%) were the smallest, implying that machine learning and ideotype-based distance capture different performance dimensions than mixed-model indices. Nevertheless, the high TCI between Pheno and LASSO (92.0%) and between Pheno and MFIDI (84.0%) indicates that the check-based method aligns closely with both machine learning approaches, likely because all three favor families that excel in multiple traits.

## 4. Discussion

### 4.1. Genetic Parameters and Variance Components

The significant block effects confirm that the augmented block design was effective in reducing environmental noise, a key advantage of this design for early-stage trials with unbalanced family sizes [7,20]. Partitioning the entry variation into orthogonal contrasts (checks, test families, and checks-vs-test) revealed that test families exhibited significant genetic variation for all traits. This partitioning is essential because when test families are compared directly against checks without this adjustment, intermediate check values can mask true genetic differences among families [7,16].

The REML/BLUP mixed model was used to estimate variance components, heritability, and genetic advance because it properly accounts for the unbalanced family sizes and the single replication of test families inherent to augmented designs [5,7]. Under such unbalanced conditions, BLUP is preferred over BLUE because it shrinks estimates of small families toward the population mean, thereby reducing bias and providing more reliable rankings for selection [10,21]. The heritability estimates obtained from BLUP indicate that productivity-related traits such as total plants per row, millable cane, and cane yield are under moderate genetic control, making them suitable targets for early-generation family selection. This agrees with previous studies that reported moderate to high family-mean heritability for yield components in sugarcane [13,22]. The moderate heritability for Brix is consistent with reports that sugar-related traits are less heritable than yield traits at the individual level but become more heritable when evaluated on a family-mean basis [23,24].

Genetic advance (GA%) followed a similar pattern: higher expected gains were observed for total plants, millable cane, and cane yield, whereas Brix and stalk height showed lower expected progress. This suggests that direct selection for Brix alone may be slow, but when combined with yield traits in multi-trait selection indices, it contributes effectively to overall selection without requiring high individual heritability [2,11,17].The use of BLUP for family ranking and selection in augmented designs has been successfully applied in several sugarcane breeding programs [4,7,13,25]. The consistency of our results with these studies confirms that REML/BLUP provides realistic, unbiased genetic parameters for early-stage selection and that productivity-related traits are the most promising targets for improving genetic gain in this population.

### 4.2. Selection Methods

#### 4.2.1. Pheno

Direct comparison with check varieties proved effective for early-stage discrimination, though its efficiency varied strongly across traits. Most families were inferior to checks in stand establishment and tillering capacity, reflecting a typical early-generation bottleneck where environmental noise suppresses these traits [13]. Wider genetic variability for stalk traits suggests stronger genetic control of plant architecture [24], while near-check Brix performance indicates feasible quality improvement. Highly skewed cane and sugar yield distributions underscore the well-known biomass-sucrose trade-off. Nevertheless, a few families (exemplified by F85) exceeded the best check in multiple traits, demonstrating that transgressive segregation can produce superior recombinants that merit multi-environment testing [4]. The comparison matrix heatmap confirmed that no single family excelled across all traits, highlighting the need for multi-trait strategies. Families with more “greater than” cells combined superior yield-related performance with acceptable quality, whereas those with none were consistently inferior, making the heatmap a reliable culling tool. Thus, phenotypic check-based selection is a practical first step, but its limitations (e.g., equal trait weighting) suggest it should be complemented by more sophisticated methods for final decisions.

#### 4.2.2. CI3

The CI3 index, based solely on cane yield, sugar yield, and millable cane, effectively discriminated performance classes. Its near-normal distribution indicated unbiased capture of genetic variation, while the slight negative skew reflected the expected difficulty of achieving high yield-related values. A key practical advantage is its simplicity: requiring only family means and z-scores, it is accessible to programs with limited statistical resources. The narrow interquartile range of the weak class allows confident culling, making CI3 a reliable first-pass screening tool that strongly agrees with BLUP for top rankings, and thus reduces the number of families needing intensive evaluation. Nevertheless, its limitations (ignoring G × E, equal economic weights, and no pedigree) mean it should be complemented by BLUP for final decisions. Integrating stability measures could further enhance its accuracy across environments [26].

#### 4.2.3. BLUP and BLUE

The combined BLUP index clearly separated elite from weak families, with the largest differences for yield-related traits, indicating substantial genetic variation. The narrower gap for stalk diameter and Brix reflects the yield-sugar trade-off. Nevertheless, elite families still achieved high Brix alongside good yield, demonstrating that combining both attributes is possible. Wide interquartile ranges among elite families justify further stringent selection, while narrow ranges in weak families allow confident culling. The top 10 families showed strong BLUP–BLUE agreement, confirming the index’s robustness. Their complementary trait profiles (high Brix in F31/F63, high cane yield in F71) offer opportunities for targeted crosses [25].

BLUP’s shrinkage provides conservative predictions for less consistent families, a valuable adjustment when environmental noise obscures genetic potential [21,27]. High heritability of yield and quality traits suggests additive gene action, facilitating early selection [4]. When comparing methods across all families, BLUP and BLUE distributions agreed closely, but BLUP identified a larger number of elite families with high index values and exhibited a more right-skewed distribution, reflecting its differential shrinkage. For index values below zero, both methods gave nearly identical results, so estimator choice does not affect culling. Therefore, BLUP is preferred for early-generation selection, while BLUE remains a valid but less discriminative alternative [10]. Given the single-location evaluation, multi-environment validation is needed [28].

#### 4.2.4. Tiered

The tiered strategy focuses on promising families while discarding poor performers early. This approach is supported by the high family-mean heritability of yield-related traits reported in the literature [22,23], which makes early-generation family evaluation a powerful filter. The fact that most selected clones originated from the top 16% of families, while discarding 68% of families, did not result in the loss of any elite clones, confirms that genetic merit is highly concentrated in a small fraction of families. This finding aligns with previous studies that demonstrated the superiority of family-based selection over individual selection in terms of resource efficiency and genetic gain [13,29]. Consequently, tiered selection provides a practical and cost-effective entry point for early-generation sugarcane breeding, especially when combined with BLUP-based ranking [26,28]. The observed selection rate falls within the typical range for early-generation breeding [9,13], indicating that the strategy is both realistic and readily applicable.

#### 4.2.5. LASSO

Integrating LASSO-based variable selection with cumulative logistic regression provided a robust framework for family-level selection. Steep sigmoidal curves for yield traits confirmed them as primary drivers of superior classification, while flat curves for Brix and stalk diameter suggested lower discriminatory power in family-based selection due to higher within-family environmental variance [9]. Thus, greater weight should be assigned to yield traits at the family evaluation stage [13]. The LASSO model achieved excellent classification (AUC = 0.95) with balanced sensitivity (0.90) and specificity (0.94), key for tiered selection: high specificity avoids misclassifying poor families as “Good”, while moderate sensitivity captures most superior families [29]. Deriving probability thresholds from cumulative logistic curves (e.g., standardized cane yield >0.6 corresponds to >80% selection confidence) offers a practical, threshold-based culling tool [14,28]. A LASSO logistic model trained on 100 families can classify families based on genotypic values before intensive phenotyping. When integrated with tiered selection, this framework efficiently discards low-performing families early, concentrating resources on promising material [10]. Future work should incorporate genomic markers to enable genomic-enabled family selection [28].

#### 4.2.6. MFIDI

The factor analysis biplot revealed MFIDI values with the top five families (F4, F31, F84, F3, and F53), four of which also ranked highly in BLUP/BLUE, confirming their multi-trait merit. Factor analysis (varimax) explained 73.9% of variance: Factor 1 was associated with yield traits, and Factor 2 with stalk diameter and Brix, suggesting simultaneous yield quality improvement without strong trade-offs [24]. The top five families lay in the positive quadrants of both factors, indicating balanced superiority. Notably, F71 (second in BLUP) did not appear in the top five because MFIDI penalizes families outstanding in a few traits but mediocre in others, promoting balanced improvement [11]. Thus, MFIDI serves as a valuable complement to BLUP, especially when targeting broad adaptation [12]. The identified families are strong candidates for further clonal evaluation and crossing.

### 4.3. Method Agreement and Consistency

Pairwise agreement among the seven selection methods confirmed that BLUP and BLUE ranked elite families similarly, with BLUP being advantageous for unbalanced data and BLUE being computationally simpler [5]. CI3 also agreed well with BLUP, indicating that three yield components capture most information needed to identify elite families without requiring pedigree or complex software [9,30]. Tiered selection showed moderate agreement for top families but very high agreement for bottom families, reflecting its efficiency in culling poor performers. LASSO and MFIDI exhibited lower agreement with BLUP, indicating they capture different dimensions of family performance, being more sensitive to ideotype definition and trait correlations. However, their high agreement with Pheno suggests they share a preference for families excelling in multiple traits. Jaccard indices were consistently lower than TCI values, providing a more conservative estimate of agreement. In conclusion, BLUP remains the gold standard as it accounts for pedigree and unbalanced data [5,30]. When computational resources are limited, BLUE or CI3 offer excellent alternatives. Tiered selection is valuable for culling poor families, while LASSO and MFIDI may provide complementary insights for specific objectives. Method choice should depend on breeding stage, available data, and goal (elite selection vs. poor family elimination).

## 5. Conclusions

Based on the results from 125 sugarcane families evaluated at the seedling stage under an augmented block design, BLUP and BLUE showed high rank agreement (Pearson’s r = 0.98; TCI = 88.0%). This confirms that both mixed-model estimators produce consistent family rankings. However, BLUP exhibited a slight shrinkage advantage, identifying more elite families with extreme index values. Furthermore, families F71, F31, and F4 consistently ranked among the top performers across most selection methods, making them the most promising candidates for further evaluation and crossing. In addition, CI3 gave a simpler alternative (TCI = 80%) when resources are limited, while tiered selection culled 85 low families (62.7% of seedlings) without losing elite clones (BCI = 84%), confirming its cost-effective step-down strategy. Moreover, LASSO achieved excellent predictive performance (AUC = 0.95), identifying cane yield, sugar yield, and millable cane as the most informative traits for distinguishing elite families. MFIDI promoted balanced multi-trait selection, with top families (F4, F31, F84, F3, and F53) excelling in both yield and quality. Based on these findings, the study suggests that BLUP with a multi-trait index can be recommended for early-generation family selection, complemented by agreement indices and LASSO to accelerate genetic gain. When resources are limited, BLUE or CI3 are excellent alternatives (>80% TCI), while tiered selection is valuable for culling poor families and MFIDI for balanced improvement.

## Figures and Tables

**Table 1:** Family codes (F1–F125), pedigree names, and number of test seedlings for the 125 sugarcane families evaluated in this study.

Code	Family	Seedlings	Code	Family	Seedlings	Code	Family	Seedlings	Code	Family	Seedlings
F1	Yuetang00-236 × ROC22	200	F32	Guitang97-40 × Yunrui03-315	150	F63	Guitang94-119 × Yacheng06-61	100	F94	Yunrui10-1288 × Yunzhe05-51	14
F2	Yacheng 07-71 × Guitang 96-211	175	F33	Zhanzhe92-126 × CP72-1210	150	F64	Zhanzhe90-76 × ROC22	100	F95	Yunrui10-299 × Dezhe99-36	14
F3	CP70-1133 × ROC22	162	F34	Funong02-3924 × Funong91-4621	150	F65	Yuegan34 × Yunrui11-111	100	F96	Yunzhe05-49 × Yunzhe08-2043	14
F4	CP72-1210 × Guitang96-211	150	F35	Funong94-0403 × Yuetang93-159	150	F66	Yuetang00-318 × Liucheng03-182	100	F97	Yunzhe99-124 × Funong90-1022	14
F5	FR96-29 × Yunrui10-336	150	F36	Funong95-1702 × ROC22	150	F67	Yuetang00-319 × CP72-1210	100	F98	Liucheng03-1137 × Yunrui10-1329	14
F6	Q72 × Yunrui 05-704	150	F37	Funong95-1702 × Zhanzhe74-141	150	F68	Yuetang03-373 × Liucheng05-291	100	F99	Liucheng05-136 × Yunrui10-1241	14
F7	RB72-454 × ROC16	150	F38	Yuenong73-204 × Dezhe93-88	150	F69	Yuetang91-976 × CP84-1198	100	F100	Funong99-20169 × Yunrui09-44	14
F8	ROC22 × Yacheng07-71	150	F39	Yuenong73-204 × Guitang02-901	150	F70	Yuetang93-159 × ROC10	100	F101	Yuetang03-393 × Yuetang89-113	14
F9	ROC22 × Yacheng00-122	150	F40	Yuetang85-177 × Guitang96-211	150	F71	Eros × Yunrui03-315	75	F102	Mintang01-77 × Yunrui11-139	14
F10	ROC24 × Yunzhe89-351	150	F41	Yuetang93-159 × ROC22	150	F72	ROC22 × Yuetang91-976	75	F103	Q96 × Yunrui06-2416	12
F11	ROC25 × Yuetang93-124	150	F42	Yuetang93-159 × Guitang94-119	150	F73	ROC28 × Yuetang89-113	75	F104	ROC22 × HoCP00-2218	12
F12	ROC26 × Yuetang91-976	150	F43	Yuetang94-128 × ROC22	150	F74	Yunzhe02-588 × ROC22	75	F105	Yunkai07-98 × Yunrui10-288	12
F13	TC7 × Yunrui06-4806	150	F44	Yuetang96-86 × ROC22	150	F75	Yunzhe94-375 × HoCP05-902	75	F106	Yunrui05-704 × Yunrui05-690	12
F14	UT1 × Yunrui10-336	150	F45	Yuetang96-86 × Yuetang89-113	150	F76	Liucheng05-291 × Liucheng03-182	64	F107	Yunrui09-311 × Yuegan40	12
F15	Yunkai07-49 × Yunrui11-111	150	F46	Yuetang99-66 × ROC22	150	F77	FR96-29 × Yunrui05-704	50	F108	Yunrui10-1229 × Yunrui09-928	12
F16	Yunrui09-28 × Yunzhe03-422	150	F47	ROC25 × Yacheng97-24	125	F78	Yunzhe02-588 × Yacheng06-92	50	F109	Yunrui10-1237 × Yunzhe05-51	12
F17	Yunrui09-44 × Yunrui03-315	150	F48	Yunzhe02-588 × Guitang02-901	125	F79	Yunzhe02-588 × Liucheng03-182	50	F110	Yunrui10-1252 × Yunrui05-780	12
F18	Yunzhe00-45 × Yunrui06-4806	150	F49	Neijiang03-218 × HoCP05-902	125	F80	Liucheng05-291 × Yuetang89-113	50	F111	Yunrui10-1252 × Yunrui09-83	12
F19	Yunzhe03-194 × Yunrui11-111	150	F50	Guitang89-5 × ROC22	125	F81	Guitang05-3595 × Guitang02-208	50	F112	Yunrui10-1288 × Liucheng03-1137	12
F20	Yunzhe94-375 × Yuetang93-159	150	F51	Zhanzhe90-76 × Guitang73-167	125	F82	Yuetang93-159 × Funong94-0403	50	F113	Yunrui10-299 × Yunzhe05-51	12
F21	Yunzhe99-601 × ROC22	150	F52	Yuetang99-66 × Funong95-1702	125	F83	Yuetang96-86 × CP89-2143	50	F114	Yunrui10-336 × Dezhe03-68	12
F22	Neijiang03-218 × HoCP01-517	150	F53	Pma98-40 × Yunrui05-704	100	F84	Q199 × Yunrui10-336	25	F115	Yunrui10-736 × Yunrui06-3504	12
F23	Yacheng05-164 × ROC22	150	F54	Pma98-44 × Yunrui06-4806	100	F85	Yunzhe99-601 × Guitang00-122	25	F116	Yunrui99-113 × UT1	12
F24	Yacheng07-71 × HoCP01-517	150	F55	ROC22 × Yuetang00-236	100	F86	Guitang73-167 × Yuetang93-159	25	F117	Yunrui99-113 × Ya93-25	12
F25	Yacheng07-71 × ROC22	150	F56	Yunkai03-206 × Yunrui05-704	100	F87	Zhanzhe74-141 × CP72-1210	25	F118	Yunzhe06-407 × Yunrui08-1276	12
F26	Yacheng93-25 × Yunrui06-4806	150	F57	Yunrui09-44 × Yunzhe03-422	100	F88	Funong02-6427 × Yuetang89-113	25	F119	Yunzhe08-2138 × Mex105	12
F27	Dezhe93-88 × ROC22	150	F58	Yunrui11-76 × Yunzhe03-422	100	F89	Yuetang00-236 × Yuetang89-113	25	F120	Yunzhe99-124 × Yunrui04-1051	12
F28	Guitang02-901 × ROC22	150	F59	Neijiang86-117 × Yuetang91-976	100	F90	Yuetang03-373 × Yuetang89-113	25	F121	Yunye06-88 × Phili63-17	12
F29	Guitang92-66 × ROC22	150	F60	Liucheng05-291 × Yuetang01-71	100	F91	Guitang94-38 × Yuetang00-236	24	F122	Funong99-20169 × Meiyin-8	12
F30	Guitang94-119 × ROC22	150	F61	Guitang00-122 × Yuetang89-113	100	F92	US84-1406 × Yunrui09-751	14	F123	Yuenong73-204 × ROC28	12
F31	Guitang94-119 × Yuetang93-159	150	F62	Guitang02-901× Ke5	100	F93	Yunrui09-926 × Yunrui10-1182	14	F124	Yuetang93-159 × ROC28	12
NA	Total of seedlings	10,955							F125	Yuetang93-159 × Yunzhe07-49	12

Full pedigree information: Chinese germplasm via YSRI (YAAS); international germplasm (CP, HoCP, FR, Q, RB, US) via USDA-ARS GRIN-Global.

**Table 2:** Structure of ANOVA for the augmented block design (Design II) used in this study.

Source of Variation (SOV)	df	SS	MS	EMS
Blocks	*b* − 1 = 3	*SS_B_*	*MS_B_*	*σ^2^_e_* +*rσ*^2^*_B_*
Entries	*n* − 1 = 126	*SS_E_*	*MS_E_*	*σ*^2^*_e_* + *g*(*σ*^2^)
Checks	*c* − 1 = 1	*SS_C_*	*MS_C_*	*σ*^2^*_e_* + *rcσ*^2^*_C_*
Test families	*g* − 1 = 124	*SS_G_*	*MS_G_*	*σ*^2^*_e_* + *rgσ*^2^*_G_*
Checks vs. Test	1	*SS_CvT_*	*MS_CvT_*	*σ*^2^*_e_* + *rcvσ*^2^*_CvT_*
Error	(*b* − 1)(*c* − 1) = 3	*SS_Err_*	*MS_Err_*	*σ* ^2^ *_e_*
Total	*N* − 1 = 291	*SS_T_*		

**Table 3:** Analysis of variance (ANOVA) for the eight agronomic traits evaluated in 125 sugarcane families across four blocks.

Source	df	Surviving Clumps	Total Plants	Stalk Height	Stalk Diameter	Brix%	Millable Cane	Cane Yield	Sugar Yield
Blocks	3	307.2 **	6552.5 **	3856.6 **	1.44 **	31.72 **	9948.0 **	10,682.0 **	234.5 **
Families (entries)	126	15.44	699.9 **	1005.1 **	0.15 **	3.24	1090.7 **	1116.0 **	21.28 **
Checks	1	21.13	1485.1 *	496.1	0.03	0.01	198.0	372.4	10.47
Test families	124	21.01 *	842.1 *	924.6 *	0.14 **	3.39	1315.8 **	1094.6 **	20.25 **
Checks vs. test	1	152.61 *	31.5	9351.9 **	2.22 **	11.57 *	1122.6 **	27,391.0 **	667.1 **
Error	3	2.38	19.45	69.87	0.00	0.39	7.00	38.90	0.56

Note: Values are mean squares. Significance levels: * *p* < 0.05, ** *p* < 0.01.

**Table 4:** Variance components, heritability, and genetic advance for eight agronomic traits estimated using the BLUP mixed model.

Trait	Model	*σ* ^2^ *g*	σ^2^e	σ^2^p	*h*^2^ (%)	GA (%)	CVe (%)	CVg(%)
Surviving clumps per row	BLUP	2.69	8.47	11.16	24.1	12.8	27.36	15.6
Total plants per row	BLUP	225.68	194.52	420.20	53.7	34.9	24.49	25.8
Stalk height (cm)	BLUP	177.05	567.80	744.85	23.8	4.7	12.01	6.8
Stalk diameter (cm)	BLUP	0.04	0.05	0.09	46.7	7.5	8.74	7.8
Brix (%)	BLUP	0.82	1.37	2.18	37.5	3.9	5.90	4.5
Millable cane (×1000/ha)	BLUP	352.62	303.93	656.55	53.7	34.9	24.72	27.7
Cane yield (t/ha)	BLUP	266.82	316.01	582.83	45.8	30.5	27.58	27.9
Sugar yield (t/ha)	BLUP	4.27	6.84	11.11	38.5	26.3	31.59	25.8

Note: *σ*^2^g: genotypic variance; σ^2^e: residual variance; σ^2^p: phenotypic variance; *h*^2^: broad-sense heritability; GA%: genetic advance as a percentage of the mean, coefficient of genetic variation (CVg).

**Table 5:** Top ten (Good) and bottom ten (Poor) sugarcane families based on the CI3 combined index, with standardized scores for cane yield (Z_Cane), sugar yield (Z_Sugar), and millable cane (Z_Millable).

Group	Rank	Family Code	Combined Index	Z_Cane	Z_Sugar	Z_Millable
Good	1	F71	2.76	2.70	2.06	3.54
Good	2	F31	1.93	1.83	2.59	1.38
Good	3	F16	1.78	1.20	1.61	2.52
Good	4	F32	1.42	1.79	0.75	1.73
Good	5	F4	1.40	1.47	2.21	0.53
Good	6	F35	1.40	1.25	1.75	1.19
Good	7	F6	1.40	1.55	1.69	0.96
Good	8	F37	1.36	1.49	0.74	1.86
Good	9	F77	1.34	1.20	1.46	1.36
Good	10	F54	1.33	1.65	0.99	1.36
Poor	116	F79	−1.36	−1.48	−1.31	−1.28
Poor	117	F64	−1.61	−1.64	−1.64	−1.55
Poor	118	F51	−1.80	−1.88	−2.11	−1.41
Poor	119	F72	−1.87	−1.88	−2.03	−1.70
Poor	120	F28	−1.88	−2.04	−1.93	−1.66
Poor	121	F78	−1.99	−2.06	−2.02	−1.90
Poor	122	F90	−2.07	−2.12	−1.93	−2.15
Poor	123	F12	−2.09	−2.13	−2.33	−1.82
Poor	124	F60	−2.12	−2.10	−2.38	−1.89
Poor	125	F59	−2.25	−2.23	−2.35	−2.16

**Table 6:** Model performance metrics on the test set (25 families, 20% of total).

Metric	Value
AUC (area under the ROC curve)	0.95
Accuracy	0.92
Sensitivity (true positive rate)	0.90
Specificity (true negative rate)	0.94

## Data Availability

The original contributions presented in this study are included in the article/Supplementary Materials. Further inquiries can be directed to the corresponding authors.

## Supplementary Materials

The following supporting information can be downloaded at: https://www.mdpi.com/article/10.3390/plants15131980/s1. Supplementary Table S1. Full list of the 125 families (codes, pedigree names, rank, class).
